# An improved F98 glioblastoma rat model to evaluate novel treatment strategies incorporating the standard of care

**DOI:** 10.1371/journal.pone.0296360

**Published:** 2024-01-02

**Authors:** Velislava Zoteva, Valerie De Meulenaere, Marthe De Boeck, Christian Vanhove, Luc Leybaert, Robrecht Raedt, Leen Pieters, Anne Vral, Tom Boterberg, Karel Deblaere

**Affiliations:** 1 Department of Radiology, Ghent University, Ghent, Belgium; 2 IBiTech—Medisip—Infinity lab, Ghent University, Ghent, Belgium; 3 Physiology Group, Department of Basic and Applied Medical Sciences, Ghent University, Ghent, Belgium; 4 Department of Head and Skin, Ghent University, Ghent, Belgium; 5 Department of Human Structure and Repair, Ghent University, Ghent, Belgium; 6 Department of Radiation Oncology, Ghent University Hospital, Ghent, Belgium; Goethe University Hospital Frankfurt, GERMANY

## Abstract

Glioblastoma (GB) is the most common and malignant primary brain tumor in adults with a median survival of 12–15 months. The F98 Fischer rat model is one of the most frequently used animal models for GB studies. However, suboptimal inoculation leads to extra-axial and extracranial tumor formations, affecting its translational value. We aim to improve the F98 rat model by incorporating MRI-guided (hypo)fractionated radiotherapy (3 x 9 Gy) and concomitant temozolomide chemotherapy, mimicking the current standard of care. To minimize undesired tumor growth, we reduced the number of inoculated cells (starting from 20 000 to 500 F98 cells), slowed the withdrawal of the syringe post-inoculation, and irradiated the inoculation track separately. Our results reveal that reducing the number of F98 GB cells correlates with a diminished risk of extra-axial and extracranial tumor growth. However, this introduces higher variability in days until GB confirmation and uniformity in GB growth. To strike a balance, the model inoculated with 5000 F98 cells displayed the best results and was chosen as the most favorable. In conclusion, our improved model offers enhanced translational potential, paving the way for more accurate and reliable assessments of novel adjuvant therapeutic approaches for GB.

## Introduction

Glioblastoma (GB) is the predominant and most malignant primary brain tumor in adults. With a median survival of only 12–15 months post-diagnosis and a 5-year survival rate of 5%, its prognosis is very poor [1–3]. GB belongs to a larger class of brain tumors, glioma, which account for 80% of all malignant primary brain tumors and arise from glial stem or progenitor cells [4, 5]. Representing more than half of all gliomas, GB is classified as a grade 4 tumor according to the 2021 World Health Organization (WHO) classification, due to its highly aggressive, infiltrative, and lethal nature [1, 6]. Conventional diagnosis of GB is performed with gadolinium (Gd)-enhanced magnetic resonance imaging (MRI) and confirmed by histological analysis [1, 5]. Treatment of GB requires a multimodal approach as described by Stupp et al. in 2005, which includes surgical resection, fractionated radiotherapy (30 fractions of 2 Gy) and concomitant and adjuvant temozolomide (TMZ) chemotherapy, considered the current standard treatment for GB [7]. Nonetheless, despite this multidisciplinary approach, patient prognoses persistently remain poor, and novel therapeutic procedures are investigated to increase overall survival and provide a better life expectancy for GB patients. In this regard, to ease preclinical testing of new therapeutics, a standardized model that mimics human GB growth as closely as possible is essential. Moreover, rising evidence indicates the use of combinatorial therapeutic approaches in addition to the standard of care, because single-agent targeted therapy often leads to drug resistance, resulting in tumor recurrence [8–10].

Animal models used for testing new therapeutics should ideally incorporate (fractionated) radiotherapy and TMZ chemotherapy, reflecting the current clinical practice for treating GB. Rat brain tumor models, including syngeneic (i.e. tissue transplant between genetically identical animals) or xenogeneic (i.e. tissue transplant between animals of different species), are widely used for *in vivo* glioma studies [11, 12]. Conversely, *in vitro* models are regarded as suboptimal, due to limitations in genetic, molecular analysis, and therapeutic strategy evaluation [11]. The ideal GB model should simulate the tumor cells’ infiltrative behavior in the brain, the tumor microenvironment, and the physiology of the blood-brain barrier (BBB). One method for achieving a preclinical model for GB is through intracranial implantation of cultured glioma tumor cell lines into the brain using stereotactic surgery (i.e. orthotopic models) [9, 12, 13]. Furthermore, the tumor microenvironment in the animal model should be surrounded by immunosuppressive cells, as this is the case with human patients as well. For this reason, immunocompetent models, which maintain this environment, remain superior to immunocompromised animals [8, 9]. Unfortunately, it is important to acknowledge that despite various advancements, none of the currently available animal brain tumor models fully reflects the complexity of human GB.

The most frequently used rat GB models are the C6, 9L, and F98 gliomas, which all three have been generated by treating adult rats with a mutagenic N-nitroso compound [14]. The C6 rat glioma model lacks a syngeneic host, as it induces a high immunogenic reaction even in Wistar rats. This drawback limits its efficacy for immunotherapy and weakens the value of survival studies [12, 15]. In contrast, both the 9L and F98 glioma cell lines grow in syngeneic Fischer rats, yet the former is highly immunogenic, and spontaneous tumor rejection can confound treatment efficacy, which limits the analysis of experimental results, while the latter model is only weakly immunogenic [11, 16]. In addition, the 9L cell line has been classified as a gliosarcoma, which is currently considered a histopathologic variant of GB [17, 18]. Hence, the F98/Fischer GB rat model is the most favorable, as this host is the least immunogenic and obtains heterogeneous infiltrative tumors with a malignant and morphological character similar to human GB [12–14, 19]. Following the implantation of F98 GB cells in Fischer rats, the established tumors exhibit resistance towards the standard of care, akin to that observed in human cases, thereby further enhancing the model’s relevance [14, 19]. Unfortunately, despite all the advantages of the F98 model, it suffers from the undesired formation of extracranial and extra-axial tumor growth post intracranial GB cell implantation, a phenomenon that is also prevalent in other GB models [12–14, 20–23]. These complications are likely attributed to reflux of the tumor cell suspension through the injection route either leading to proliferation onto the skull or invasion through the cranial sutures, forming extracranial and extra-axial tumors respectively. This undesirable tumor growth is detrimental to the animals and often requires early euthanasia, making survival studies infeasible [21, 23].

In this study, our objective was to develop a highly reliable and reproducible rat model for GB that closely resembles the clinical context. To evaluate the effectiveness of novel therapeutic approaches in combination with the current standard treatment, we included the standard of care consisting of fractionated radiation therapy and TMZ chemotherapy in the rat model. By incorporating the standard of care into the rat model, we aimed to enable the evaluation of potential synergistic effects or enhanced efficacy of novel adjuvant therapies. In addition, we optimized the model by modifying the intracranial inoculation of the cancer cells and the radiotherapy protocol to eliminate extra-axial and extracranial tumor growth. To the best of our knowledge, this is the first study where the F98 GB rat model combines (hypo)fractionated radiotherapy with TMZ chemotherapy to explore the effect of the number of inoculated cells.

## Materials and methods

### Cell culture

All chemical products were purchased from Thermo Fisher Scientific (Massachusetts, United States) unless specified otherwise. The F98 cell line was obtained from the American Type Culture Collection (RRID:CVCL_3510, CRL-2397^™^, Virginia, United States). More precisely, the cell line was developed by injecting a single dose of N-ethyl-N-nitrosourea (ENU) into pregnant Fischer rats on the 20th day of gestation. The offspring developed a brain tumor that was harvested and maintained in culture. The F98 cancer cells were cultured in monolayers using Dulbecco’s modified Eagle’s medium (DMEM), supplemented with 10% Fetal Bovine Serum (FBS), 1% penicillin/streptomycin antibiotics, 0.1% Amphotericin B and 1% sodium pyruvate (100 mM, Sigma Aldrich, Missouri, United States), and maintained at 37°C and 5% CO_2_.

### Ethics statement and animal maintenance

This research protocol was approved by the Ghent University ethics committee for animal experiments (ECD 21/06). All rats were kept and handled according to The European guidelines (directive 2010/63/EU) and housed under environmentally controlled conditions (normal light/dark cycle period of 12 hours, 20°C-24°C, and 40–70% relative humidity). The animals were daily monitored and provided with food and water ad libitum.

### Experimental setup: The F98 GB rat model

#### Intracerebral implantation

Female Fischer rats (n = 28) (F344/IsoCrl, Charles River^®^; age 11 ± 1 weeks, mean ± SD; body weight 155 ± 11g, mean ± SD) were anesthetized with isoflurane (induction 5%, maintenance 2%) mixed with oxygen (0,5L/min), and immobilized in a stereotaxic frame (Model 902 Dual Small Animal Stereotaxic frame, Kopf1). Their rectal temperature was maintained at 37.0 ± 0.5 °C with a feedback-controlled heating pad connected to a rectal probe. Next, the head of the rat was shaved, following exposure of the skull through a scalp incision. A 1 mm hole was drilled (diamond drill, Dremel^®^) in the skull 8 mm posterior and 4.5 mm lateral to the bregma. Subsequently, a stereotactically guided syringe (29G × 12.7 mm) with a 5 μL PBS cell suspension containing either 500 (n = 5), 1000 (n = 6), 5000 (n = 6), 10 000 (n = 6), or 20 000 (n = 5) F98 glioma cells were injected 4.1 mm deep (right entorhinal cortex), at a rate of 0.5 μL/min, using a micro syringe pump controller (Micro 4TM, World Precision Instruments, Sarasota, USA), hence creating five experimental groups. Post-inoculation, the syringe was kept in position for 5 minutes and then, slowly, in steps of 0.7 mm and 2 minutes waiting time per step, withdrawn (total of 12 minutes in six steps). The burr hole was closed with simplex rapid powder (Kemdent^®^, Swindon, England), the skin was sutured and a subcutaneous injection of meloxicam (1 mL/kg) was administered. Subcutaneous injection of sodium Chloride 0.9% w/v and Glucose 5% w/v solution (2mL/kg) was administered to prevent dehydration. Finally, Neobacitracine (BePharBel Manufacturing, Courcelles, Belgium) was applied to the wound as a local antibiotic.

#### Confirmation of GB growth with MRI

MRI scans were performed to verify tumor development in the brain (7T μMRI, PharmaScan 70/16, Bruker BioSpin, Ettlingen, Germany). Hence, rats were anesthetized with isoflurane (induction 5%, maintenance 2%) mixed with oxygen (0.5L/min), and an intravenous injection (IV) of a Gd-based contrast agent (Gadovist^®^, Bayer, Germany; 1mmol/kg) was administered into a tail vein. Then, the rat was fixated on a heating pad in the restrained bed, while the body temperature was maintained at 37°C. A rat brain surface coil (Rapid Biomedical, Rimpar, Germany) was secured around the head and the bed was positioned in a 72 mm rat whole-body transmitter coil (Rapid Biomedical, Rimpar, Germany). To localize the tumor, measure tumor volumes, and demonstrate the increased permeability of the BBB present in GB, a contrast-enhanced (CE) T1-weighted (T1-w) spin echo sequence (117 μm in-plane resolution, TR/TE 1539/9.7 ms, 3 averages, TA 4’15”) was obtained. MRI scans were initiated five days post intracerebral implantation, followed by scans every two days until the tumor’s diameter reached 2.5–3.5mm. Upon this occurrence, GB growth was confirmed, designating this day as day 0.

#### Treatment protocol

The day after tumor confirmation (i.e., day 1), all rats received standard treatment consisting of fractionated MRI-based radiotherapy and concomitant TMZ chemotherapy.

#### Hypofractionated MRI-based radiotherapy (3 x 9 Gy)

**Fig 1:**
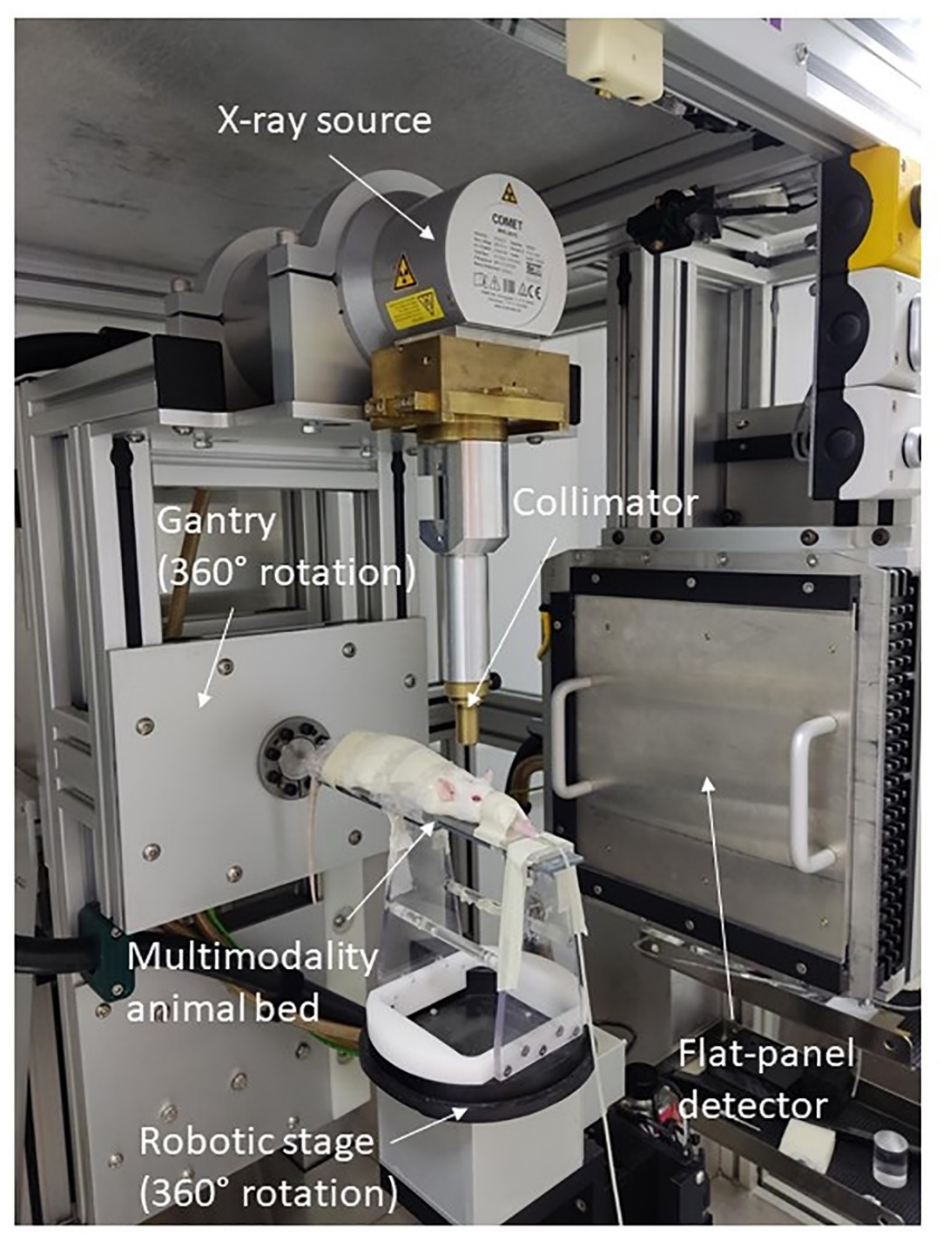
Visualization of the preclinical image-guided microirradiator SARRP (small-animal radiation research platform) with an F98 Fischer GB rat.

The MRI-guided radiotherapy treatment was performed using the SARRP (Fig 1) (XStrahl, United Kingdom). First, the rat was anesthetized with isoflurane (induction 5%, maintenance 2%) mixed with oxygen (0.5L/min), and an IV injection of Gadovist^®^ was administered into a tail vein. Next, the rat was fixed on a multimodality bed, and a CE T1-w MRI scan was performed. Finally, the rat was transferred to the four-axis robotic positioning table of the SARRP.

**Fig 2:**
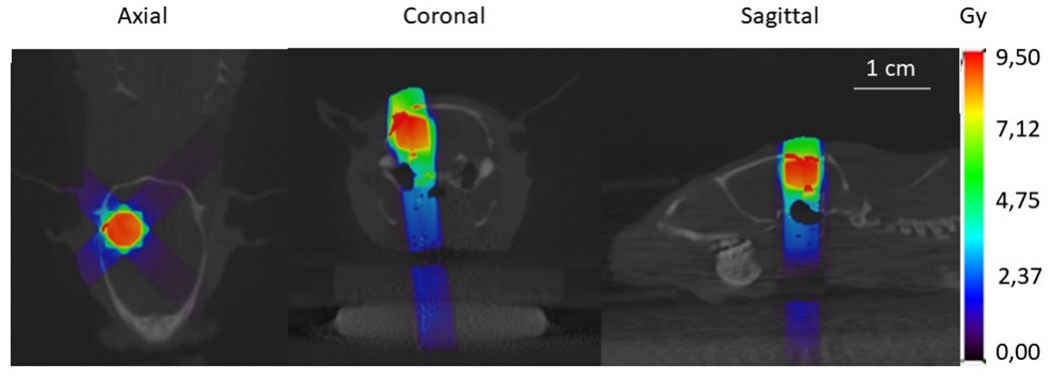
Visualization of the radiation dose distribution on the CT image. Axial, coronal and sagittal views, using two non-coplanar arcs and one static beam parallel and following the inoculation track to deliver three fractions of 9 Gy to the target GB volume, delineated on Gd-enhanced T1-w MRI.

A treatment planning CT was obtained with a total of 720 projections acquired over 360° (voltage X-ray source: 60 kV, tube current: 600 μA, and aluminum filter: 1 mm). Subsequently, the CT data were reconstructed using an isotropic voxel size of 0.2 mm and imported into the treatment planning software Muriplan (Version 3.0.0, Xstrahl, UK) to distinguish air, soft tissue, and bone by manual segmentation. The obtained MR image was precisely co-registered with the planning CT and the center of the contrast-enhanced tumor region on the CE T1-w MR image was selected as the target for irradiation (i.e., isocenter). Using a 5 x 5 mm collimator, a total dose of 27 Gy was delivered in 3 fractions of 9 Gy (voltage X-ray source: 220kV; tube current: 13 mA; copper filter of 0.15 mm) on day 1, day 3, and day 5, by applying a single beam parallel and following the inoculation route, and two non-coplanar arc beams (arc rotations of 120°). In Fig 2, the radiation dose distribution of one fraction of 9 Gy in one of the animals is demonstrated. 3 fractions of 9 Gy are roughly equivalent to 60 Gy in 2 Gy fractions, which is the clinically applied dose to treat GB in humans.

#### TMZ chemotherapy

Each rat received, concomitantly with the radiotherapy, IP injections of TMZ (29mg/kg; MedChem Express, New Jersey, United States), dissolved in 20% DMSO (Sigma Aldrich, Missouri, United States) and diluted with saline to 1mL for five consecutive days (i.e., day 1–5). Before the injection, the rat was anesthetized with isoflurane (induction 5%, maintenance 2%) mixed with oxygen (0.5L/min).

#### MRI follow-up

**Fig 3:**
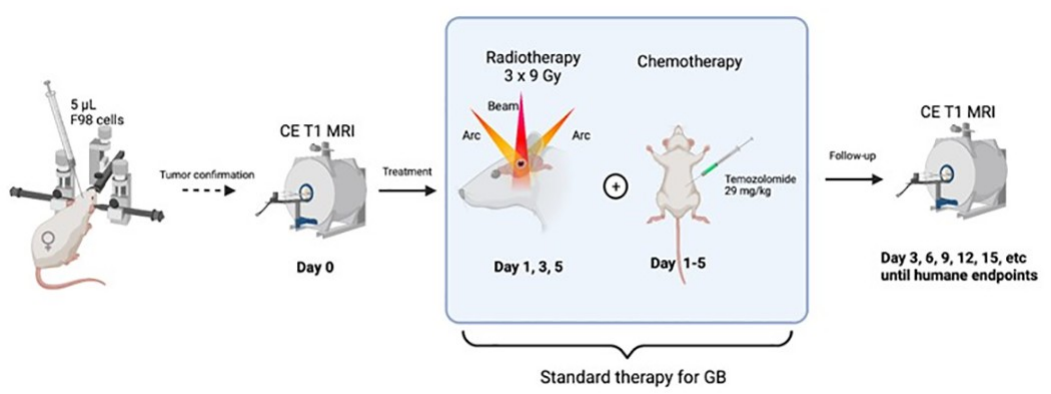
The total experimental setup for the development of the F98 Fischer GB rat model. Starting with the intracranial inoculation of F98 GB cells in the right entorhinal cortex. After a certain amount of days, depending on the number of cells inoculated, GB growth was confirmed with Gd-enhanced T1-w MRI (i.e. day 0), followed by the treatment protocol (starting from day 1). The latter consists of fractionated radiotherapy (3x9 Gy) and TMZ chemotherapy. Finally, tumor growth was monitored with MRI until humane endpoints were reached (Created with BioRender.com).

To evaluate treatment response, CE T1-w MRI sequences were obtained every three days until humane endpoints were reached (i.e., day 3, day 6, day 9, …) and tumor volumes were measured (see below). An overview of the full experimental setup is shown in Fig 3.

#### Euthanasia: Humane endpoints

During the experimental setup, all rats were daily examined for specific clinical and behavioral signs of distress, including balance problems, diminished activity, lack of grooming, weight loss (a reduction of more than 20% of the weight), and/or a hunchback posture. Additionally, the CE T1-w MRI scans were monitored for GB growth (euthanasia when tumor volume was >40% of total brain volume) and pathological changes, including the invasion of a complete cerebral hemisphere, extreme growth of an extra-axial or extracranial tumor, midline shift, or expansion of the ventricles. When humane endpoints were reached, instantaneous euthanasia was performed by an IV injection of natrium pentobarbital (100 mg/kg; Euthanimal 20% (200 mg/mL); Hoogstraten, Belgium). An overview of the humane endpoints for each rat can be found in the supplementary data (S1 Table).

### Histological analysis

The brain of one rat of each experimental group was isolated upon euthanasia. Next, the brain was immersed for 24h in 4% paraformaldehyde and embedded in paraffin. Finally, the cerebrum was sectioned in slices of 5 μm and stained with hematoxylin and eosin (H&E). The presence of GB tumor, the pattern of infiltration, and intrinsic tumor characteristics were investigated.

### Data and statistical analysis

Tumor volumes were calculated on the CE T1-w MR scans, using the Fiji software, by manually outlining the enhancing regions on individual slices and multiplying the areas by the slice thickness (0.6 mm). Statistical analysis of the tumor volumes on day 0 between the 5 groups is performed using the Kruskal-Wallis test. Statistical analysis of the GB tumor volumes at the different time points between the five groups is performed using mixed model analysis with Bonferroni correction for multiple comparisons. Next, binary scoring for extracranial tumor presence (classification: present/absent) on the CE T1-w MR images of each animal was performed. Statistical analysis of the extracranial tumor growth prevalence between all groups was performed using Fisher’s exact test. In addition, binary scoring for extra-axial tumor formation was performed (classification: favorable/unfavorable) on the CE T1-w MR images and compared between different rat groups. Logistic regression was used to estimate odds ratios, and a Cox proportional hazards model was performed for survival analysis for time-to-event outcomes (event = reaching humane endpoints). A probability value of p < 0.05 is considered statistically significant. Statistical tests were executed either with GraphPad Prism version 8.4.2 or RStudio version 3.5.2 (source code available at https://github.com/VelislavaZoteva/PlosOne.git).

## Results

### Effect of the number of inoculated cells

#### Effect on tumor confirmation

**Fig 4:**
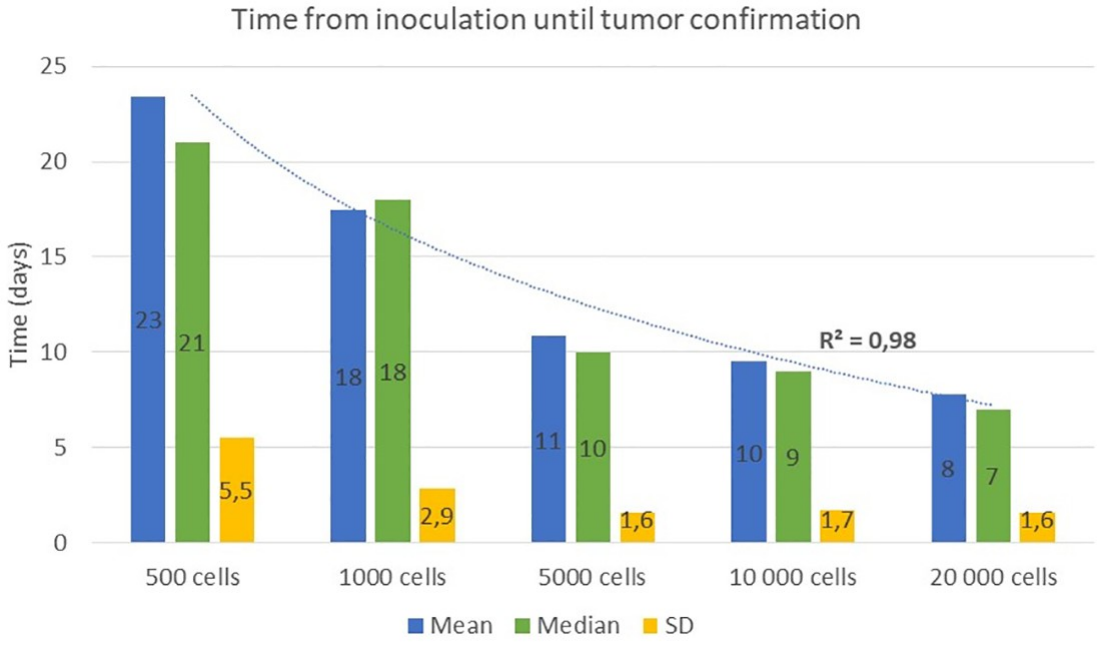
The number of days until tumor confirmation is inversely proportional to the amount of inoculated F98 cells in the five rat groups. A negative correlation is found between the number of F98 GB cells inoculated and the number of days until tumor confirmation (R^2^ = 0.98). The SD, median and mean values of the days until tumor confirmation are shown per experimental group. A decrease in SD values is observed as the cell amount increases.

All animals (100%) developed a brain tumor at the inoculation site over a period that varied according to the number of inoculated GB cells. As anticipated, the time interval from the inoculation procedure to the confirmation of GB demonstrated an inverse correlation with the number of implanted cells (characterized by a logarithmic decline, R^2^-value = 0.98). The mean, median and SD values of the days between inoculation and tumor confirmation for all groups are graphically represented in Fig 4. Generally, the time until confirmation of GB growth of the groups inoculated with 500 and 1000 F98 GB cells show larger SD values (5.5 and 2.9 days respectively), compared to the groups inoculated with a higher number of cells (i.e. 5000 (1.6 days), 10 000 (1.7 days) and 20 000 (1.6 days) cells). The mean tumor volumes at day 0 for the animals inoculated with 500, 5000, 10 000, and 20 000 GB cells show similar mean ± SD values: 27.26 ± 12.15 mm^3^, 18.47 ± 6.45 mm³, 25.58 ± 12.22 mm^3^ and 17.06 ± 9.79 mm^3^, respectively. The animals inoculated with 1000 GB cells display the largest variation in tumor volumes at day 0 (47.43 ± 29.70 mm^3^). The Kruskal-Wallis test revealed no statistically significant difference in tumor volume on day 0 among the five experimental groups (p-value = 0.408).

#### Effect on treatment response

**Fig 5:**
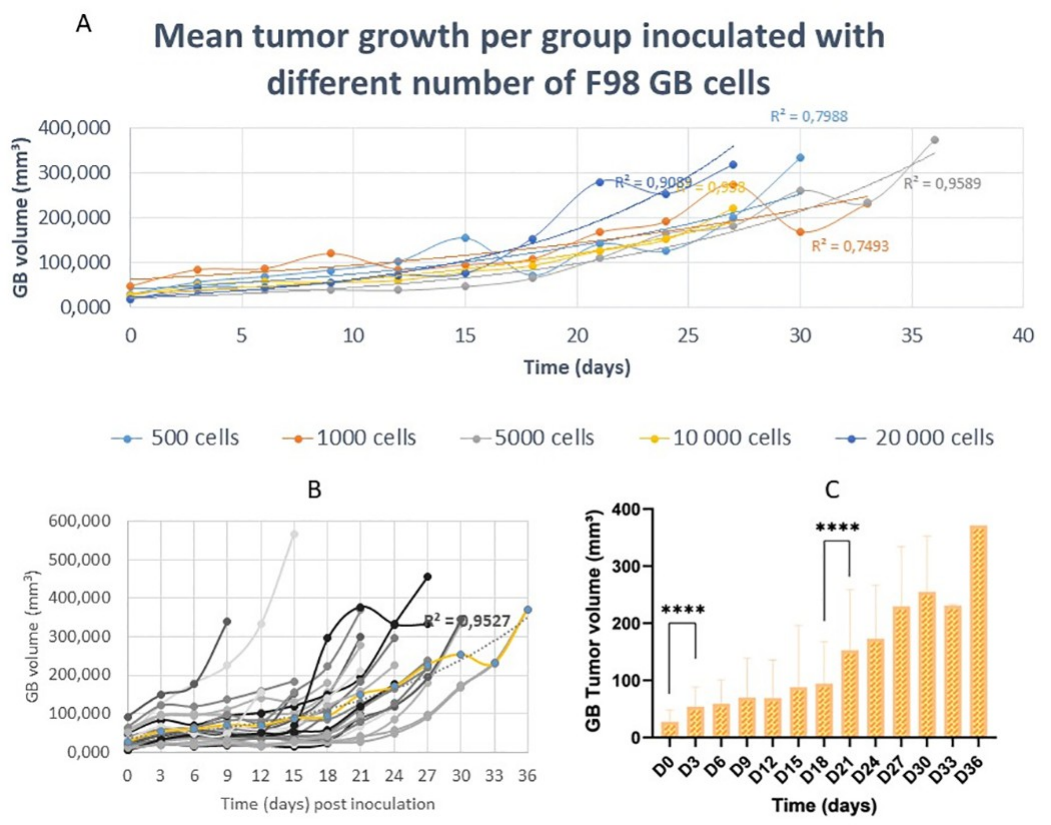
The progression of the GB tumor over time, in rats inoculated with different amounts of F98 GB cells and receiving the standard therapy (fractionated radiotherapy and TMZ chemotherapy), shows an exponential growth pattern. (A) The progression of the mean GB tumor volume per group shows an exponential growth pattern for rats inoculated with 500 F98 GB cells (R^2^ = 0.79), rats inoculated with 1000 F98 GB cells (R^2^ = 0.75), rats inoculated with 5000 F98 GB cells (R^2^ = 0.96), rats inoculated with 10 000 F98 GB cells (R^2^ = 0.96) and rats inoculated with 20 000 F98 GB cells (R^2^ = 0.91). (B) The mean GB volumes per measured time point of all groups show an exponential increase (R^2^ = 0.95, yellow line). (C) For all animals in every group, a significant difference in mean tumor volume is observed between day 0 and day 3 (p < 0.0001) and between day 18 and day 21 (p < 0.0001). Between day 3 and day 18 the mean tumor volume increases insignificantly.

The volumes of the GB tumors for all groups, treated with both radiotherapy and TMZ chemotherapy, exhibit an exponential increase over time (R^2^ = 0.95), as elucidated in Fig 5B. Fig 5C demonstrates the mean GB tumor volume of all groups at each time point. A significant difference in mean tumor volume was observed between day 0, i.e. confirmation day, and day 3 (p-value < 0.0001), as well as between day 18 and day 21 (p-value < 0.0001). The quantified mean GB tumor volumes on day 0, day 3, day 18, and day 21 correspond to 27.52 ± 19.91 mm^3^, 53.97 ± 34.01 mm^3^, 94.41 ± 71.46 mm^3^ and 152.95 ± 103.55 mm^3^, respectively. Conversely, during days 6, 9, 12, and 15, the mean GB tumor volume attains a state of stabilization, showing insignificant increases with total mean volumes of 59.16 ± 40.79 mm^3^, 70.30 ± 67.75 mm^3^, 69.25 ± 65.27 mm^3^ and 88.08 ± 106.46 mm^3^, respectively. The progression of the mean tumor volumes after treatment for each group is shown in Fig 5A. The R^2^-values after exponential regression for tumor volumes inoculated with 500 F98 GB cells, 1000 F98 GB cells, 5000 F98 GB cells, 10 000 F98 GB cells, and 20 000 F98 GB cells are, respectively, equivalent to 0.79, 0.75, 0.96, 0.96 and 0.91, which indicates that the GB has an exponential growth over time. The GB tumor volumes of each animal per timepoint for each group are visualized (see supplementary data, S1 Fig).

#### GB growth progression of a rat inoculated with 5000 F98 GB cells

**Fig 6:**
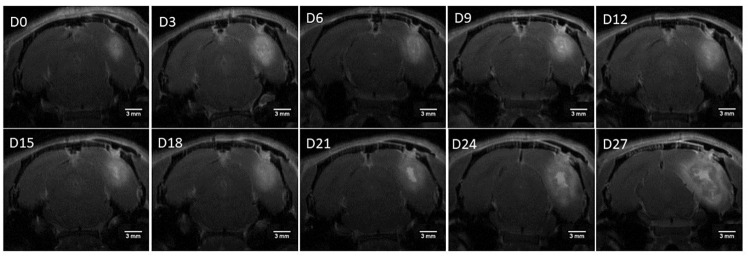
Visualisation of the GB tumor growth progression on Gd-enhanced T1-w MR images in the F98 GB rat model, inoculated with 5000 F98 cells, after receiving standard therapy (i.e. hypofractionated radiotherapy and TMZ chemotherapy). Abbreviation: day (D), with D0 is equal to day of GB confirmation. Scale bar: 3 mm.

The GB growth pattern in a rat inoculated with 5000 F98 GB cells is shown in Fig 6 at different time points using Gd-enhanced T1-w MR images. The measured tumor volumes for that specific animal were 7.11 mm^3^ (day 0); 21.45 mm^3^ (day 3); 21.76 mm^3^ (day 6); 21.65 mm^3^ (day 9); 19.81 mm^3^ (day 12); 22.41 mm^3^ (day 15); 25.59 mm^3^ (day 18); 26.84 mm^3^ (day 21); 48.28 mm^3^ (day 24) and 90.44 mm^3^ (day 27), indicating exponential growth over time. Additionally, in Fig 6 the stabilization of the GB tumor volume between day 3 and day 18 is demonstrated.

#### Histologic analysis

**Fig 7:**
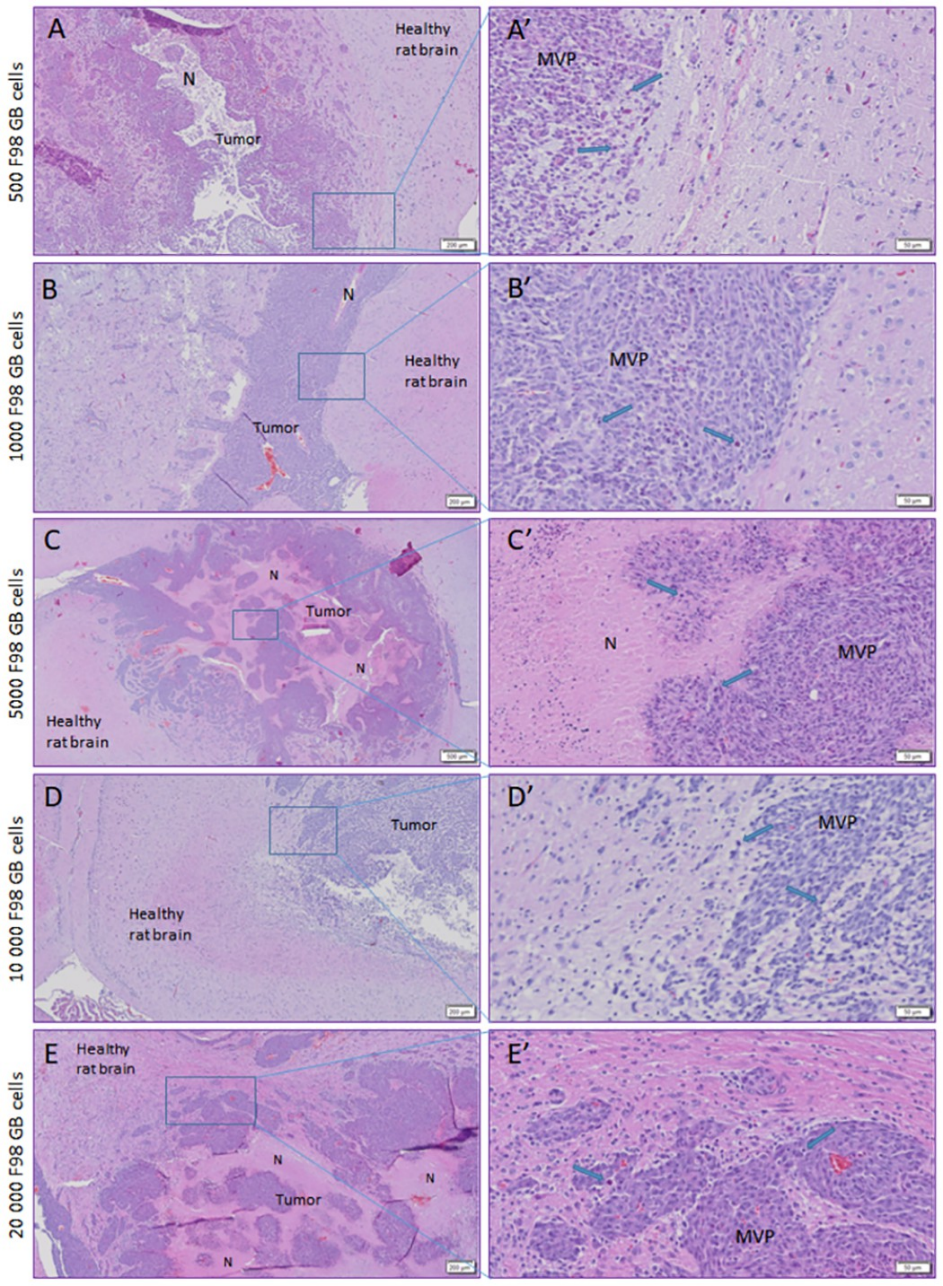
Histological features in the F98 GB rat model. H&E stained brain sections of rats inoculated with 500 F98 GB cells (A, A’), 1000 F98 GB cells (B, B’), 5000 F98 GB cells (C, C’), 10 000 F98 GB cells (D, D’) and 20 000 F98 GB cells (E, E’). The blue rectangles indicate the region, which is shown at a higher magnification, in the right image next to it. The rats displayed histological characteristics of GB: giant cell formation and nuclear pleomorphism (blue arrow), central necrotic core (N) and mircrovascular proliferation (MVP). Scale bar: 500 μm (C), 200 μM (A, B, D, E), 50 μM (A’-E’).

Histological features of GB are displayed on H&E stained brain sections of each experimental rat group (Fig 7), confirming GB tumor development as well as the predetermined implantation site, i.e. the right entorhinal cortex. Tumors showed mitotic activity and tumor infiltration in the surrounding brain parenchyma. The presence of microvascular proliferation, giant cell formation, and nuclear pleomorphism was observed in all samples. A central necrotic core was observed in each specimen. There were no visible differences in histological characteristics between the five different rat groups.

### Extra-axial and extracranial tumor formations

#### Visualisation of extracranial and extra-axial tumor formations on T1-w MRI

**Fig 8:**
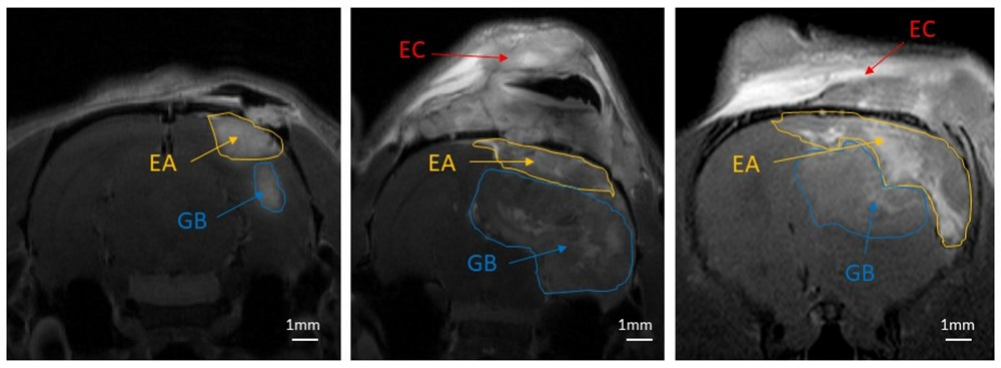
Illustration of extracranial (i.e. on the skull surface) and extra-axial (i.e. between the meninges and the skull) tumor growth on Gd-enhanced T1-w MR images. (A,A’) GB and extra-axial growth at day 0, i.e. confirmation day. (B,B’,C,C’) GB, extra-axial and extracranial tumor growth when humane endpoints were reached. Abbreviations: glioblastoma (GB), extra-axial tumor growth (EA), extra-cranial tumor growth (EC). Scale bar: 1 mm.

Fig 8 visualizes the GB tumor, as well as the unwanted extra-axial and extracranial tumor formations in the F98 GB rat model inoculated with 10 000 or 20 000 F98 cells, using Gd-enhanced T1-w MRI. Fig 8A and 8A’ represent the day of GB confirmation (i.e., day 0), where the volumes of GB and the extra-axial tumor were 9.35 mm^3^ and 30.90 mm^3^, respectively. Fig 8B, 8B’, 8C and 8C’ are Gd-enhanced T1-w MR images of two different rats showing extra-axial and extracranial tumor formations at the time of euthanasia, i.e. when humane endpoints were reached. The corresponding volumes of GB and extra-axial were 221.25 mm^3^ and 30.10 mm^3^ (Fig 8B’), and 66.00 mm^3^ and 182.99 mm^3^ (Fig 8C’), respectively.

#### Prevalence of extracranial tumor growth in rats inoculated with different numbers of F98 cells

**Fig 9:**
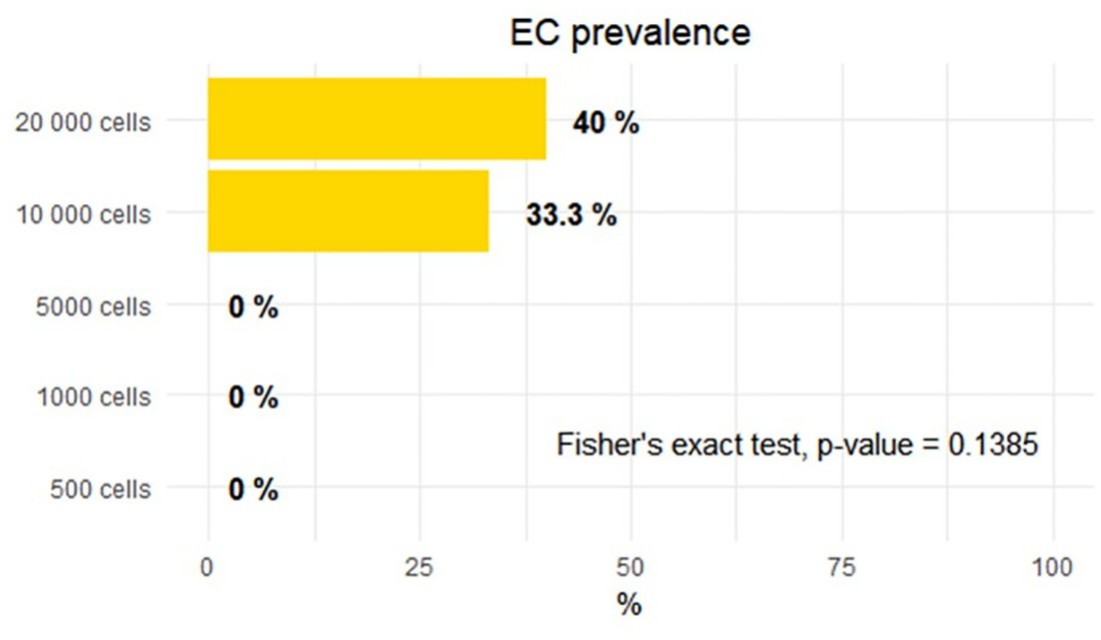
Percentages of extra-cranial (EC) tumor growth in rats inoculated with different amounts of F98 GB cells (binary scoring) shows no significance. Animals inoculated with 500 (n = 5), 1000 (n = 6) and 5000 (n = 6) cancer cells, showed no presence of extracranial tumor growth. In rats inoculated with 10 000 cells (n = 6) and 20 000 cells (n = 5), respectively 33% and 40% developed extracranial tumor growth. Fischer’s exact test showed no significant difference between the groups (p = 0.139).

All animals were investigated for extracranial tumor growth on the CE T1-w MR scans on the day the rats reached their humane endpoints. The animals inoculated with 500, 1000, and 5000 F98 cells did not develop extracranial tumor growth, but those inoculated with 10 000 and 20 000 F98 GB cells showed extracranial growth in 33% and 40% of the rats, respectively (Fig 9). These differences did however not attain statistical significance (Fisher’s exact test, p-value = 0.139).

#### Probability of extra-axial tumor formation and survival in rats inoculated with different numbers of F98 cells

**Fig 10:**
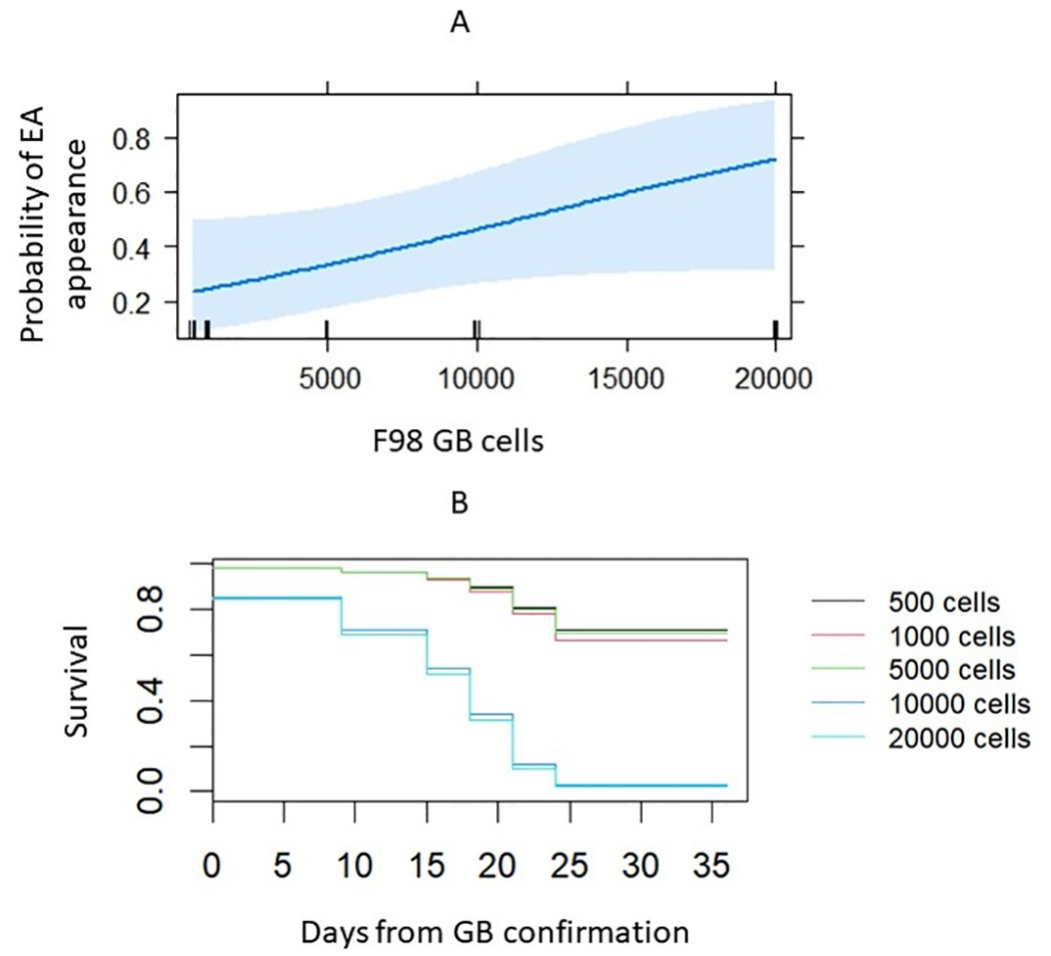
A reduction of extra-axial (EA) tumor growth and an increase in overall survival is observed as the cell amount decreases. (A) Logistic regression analysis showed that as the cell amount increases, the probability of extra-axial tumor formation increases (500 vs 20 000 F98 GB cells; p = 0.076). (B) The Cox proportional hazards model showed that the overall survival is not statistically significant between rats inoculated with 500 F98 GB cells and rats inoculated with 1000 and 5000 F98 GB cells (p-values = 0.91 and 0.97 respectively), but rats inoculated with 500 F98 GB cells had significantly better overall survival compared to rats inoculated with 10 000 and 20 000 F98 GB cells (p-values = 0.043 and 0.037 respectively).

Fig 10A shows a trend of increasing probability of extra-axial tumor growth as the inoculated cell amount increases. The risk of extra-axial tumor formation in rats inoculated with 500 F98 GB cells equals 23%, while in rats inoculated with 20 000 F98 GB cells the probability corresponds to 72%. In other words, the probability of no extra-axial tumor formation is 77% in rats inoculated with 500 F98 GB cells, compared to a probability of 28% in rats inoculated with 20 000 F98 GB cells. However, there was no significant difference in the odds of extra-axial tumor appearance between the five animal groups (OR = 0.9999, p-value = 0.076). The Cox proportional hazards model, presented in Fig 10B, shows rats inoculated with 500, 1000, and 5000 F98 GB cells had a better overall survival, compared to rats inoculated with 10 000, and 20 000 F98 GB cells. The overall survival is not statistically significant between rats inoculated with 500 F98 GB cells and rats inoculated with 1000 and 5000 F98 GB cells (p-values = 0.911 and 0.971 respectively). Rats inoculated with the lowest amount of F98 GB cells, i.e. 500 GB cells, had significantly better overall survival compared to rats inoculated with 10 000 and 20 000 F98 GB cells (p-values = 0.043 and 0.037 respectively).

## Discussion

Over the past decades, advancements in refining animal models for studying GB have been facilitated by advanced techniques, including the use of stereotaxic frames for precise tumor cell implantation, image-guided radiotherapy systems such as the SARRP, and tools for treatment evaluation and biomarker analysis, such as CE T1w MRI [24–26]. Nonetheless, challenges persist, underscoring the fact that, up to this point, none of these models fully reflects human GB. *In vivo* animal models not only improve our comprehension of GB’s pathological and molecular characteristics but also aid in the progress of testing and developing new treatment strategies. To do so, a suitable and reproducible GB animal model is of significant importance. GB is one of the most challenging tumors to treat, largely due to its exceedingly unfavorable prognosis (median survival of 15 months), a consequence of its diffusely infiltrative growth pattern and intratumoral molecular heterogeneity [6, 7]. Animal models are imperative for both evaluating innovative therapies through preclinical feasibility studies and for predicting clinical results in humans [27]. To examine novel adjuvant therapies, it is crucial to integrate the standard of care—comprising fractionated radiotherapy and TMZ chemotherapy—into GB animal models as closely aligned with the human clinical context as possible. In addition, a proficient brain tumor model should adhere to a defined set of criteria: (1) faithfully reflecting human GB in terms of tumor microenvironment and growth characteristics, encompassing intratumoral heterogeneity, neovascularization, alterations in the BBB, and an invasive growth pattern; (2) demonstrating a predictable and reproducible tumor growth rate; (3) minimizing the time interval between tumor implantation and tumor confirmation; (4) ensuring a sufficient length of survival duration in treatment evaluation studies, enabling effective determination of therapeutic efficacy; (5) manifesting non- or weak immunogenicity within syngeneic hosts; and (6) precluding the development of both extra-axial tumors (into the epidural or subdural space) and/or extracranial tumors (extending beyond the brain) [12, 26, 28].

As previously described, the most widely used C6 and 9L malignant glial cell lines display clear limitations, such as immune responses leading to tumor rejection in Wistar rats or poor infiltration into the surrounding brain tissue, respectively. It is worth noting that, within the context of animal model selection, these cell lines do showcase potential for blood-brain assessments, metabolic investigations, genetic inquiries, and *in vitro* assays. However, their utility to investigate tumor growth dynamics and responses to novel therapeutic strategies is compromised [12, 15, 26, 29]. For studies focused on prolonging overall survival with the ultimate goal to cure GB, the weakly immunogenic F98 GB rat model emerges as an optimal choice, as it meets most of the aforementioned criteria [12]. Unfortunately, the recurrent manifestation of undesired extra-axial and extracranial tumor growth within the syngeneic F98/Fischer GB rat model diminishes its clinical relevance, a phenomenon rarely encountered in human cases. Many researchers have encountered similar complications in several other glioma animal models, defining extra-axial tumors as subdural growth or intracranial extra-cerebral spread [13, 14, 20–22, 30, 31]. In addition, the F98 cells can spread to the surface of the skull, constituting extracranial tumors. Presumably, this can be attributed to the highly invasive growth pattern of F98 cells, along with the backflow of tumor cells through the needle track during the implantation procedure [22, 30]. As a result, these large space-occupying lesions necessitate euthanasia, as described by De Meulenaere et al., thereby making survival studies infeasible [21]. Hence, minimizing the extra-axial and extracranial tumor growth is of major importance, both to promote animal welfare and respect the 3Rs (Replacement, Reduction and Refinement, i.e. guiding principles for more ethical use of animals in scientific research), but also to ensure the attainment of reliable scientific results.

Therefore, in this study, the design of the F98 GB rat model is further optimized by integrating the standard of care, so that this model can be used for preclinical studies to evaluate novel adjuvant treatment strategies. More precisely, we adjusted the inoculation procedure to standardize the GB model and to minimize extra-axial and extracranial tumor growth formations. In addition, we modified the radiotherapy protocol, previously described by Bolcaen et al., to enhance the clinical relevance of the model and to suppress the progression of extracranial and extra-axial tumor growth [32]. To this end, all steps in the intracerebral inoculation procedure should be executed with the highest precision, given that each step has the potential to influence the accuracy and reproducibility of the rodent model, subsequently compromising the integrity of scientific outcomes [23, 30, 33]. The utilization of a stereotactic frame and micro-infusion pump is of major significance for obtaining accurate and consistent tumors, a technique elucidated as early as 1980 by Kobayashi et al. [23]. Tumor implantation by freehand injection resulted in poor intracerebral growth yield, often accompanied by spread to intracranial tissues, and/or an inconsistent location of the tumors. Furthermore, increased expertise in the stereotactic implantation technique markedly improved outcomes [23]. Moreover, we hypothesized that the smallest infusion volume, coupled with the lowest cell count and the slowest infusion as possible, would decrease the risk of cell suspension backflow along the implantation track, thereby decreasing the likelihood of extra-axial and extracranial tumor formations. Mathieu et al. inoculated different numbers of F98 GB cells (1x10^3^, 1x10^4^, 1x10^5^, 2x10^5^, and 5x10^5^ cells) in the syngeneic Fischer rat in three different volumes (10 μL, 5μL and 1 μL) [30]. However, since De Meulenaere et al. documented instances of extra-axial and extracranial tumor formations with the implantation of 25 000 F98 GB cells, we opted to further reduce the inoculated cell number [21]. At the lowest end of the scale, it is known that inoculating at least 100 cells is required to guarantee tumor development [13, 31]. Therefore, the Fischer rats were inoculated with cell numbers in the range of hundreds (500 F98 GB cells) to thousands (1000, 5000 F98 GB cells) and compared with rats inoculated with 10 000 and 20 000 F98 GB cells. Next, the suspension volume was set to 5 μL, considering Mathieu et al.’s findings that volumes surpassing 5 μL displayed extra-axial or extracranial tumor formations [30]. A suspension volume of 1 μL was regarded as too low, as this occasionally resulted in the absence of tumor development [30]. In the present study, tumor uptake within the F98 GB rat model was 100% for each experimental group, underscoring the pivotal role of the stereotactic frame, the micro-infusion pump, and the choice of cell suspension volume. The last modification in the inoculation procedure entails the gradual withdrawal of the syringe, facilitated in six steps. In the investigation of photodynamic therapy response in glioma, Bulin et al. utilized the F98 rat model and provided a detailed protocol for the stereotactic implantation of the F98 cells. However, the protocol did not include a waiting period before needle removal, nor did it specify gradual withdrawal of the syringe [34]. Kobayashi et al. suggested that delaying needle extraction from the brain for four to five seconds could lead to a reduction in cell backflow [23]. Moreover, Rama et al. introduced a fifteen-second waiting period before needle removal, while Vinchon-Petit et al. prolonged this interval even further to five minutes [13, 35]. Consequently, we consider the multiple waiting steps as crucial for reducing the occurrence of extra-axial and extracranial tumor formations, as this minimizes the risk of cell suspension retraction through the needle track.

As expected, the time from inoculation to GB confirmation with Gd-enhanced T1-w MRI varied according to the number of inoculated cells, demonstrating an inversely proportional correlation. Rats inoculated with 500 and 1000 F98 cells showed a larger variation in days until confirmation, compared to the groups inoculated with 5000, 10 000, and 20 000 F98 cells. Dividing the cell number by a factor of 40 (i.e., 500 cells vs 20 000 cells), approximately tripled the days until confirmation, along with the degree of variation amongst the individual animals. This should be considered before choosing the right number of cells for the specific experimental design objectives.

To mimic the clinical situation and to assess brain tumor information, such as tumor volume, tumor vascularization, BBB permeability, and responses to the fractionated radiotherapy and TMZ chemotherapy, CE T1-w MRI was used as an imaging modality [22, 36]. Moreover, histological grading was performed for GB diagnosis and to analyze the characteristics of each experimental group. The typical characteristics of GB, such as the infiltrative character along tumor margins, formation of giant cells, nuclear pleomorphism, and the presence of a central necrotic core, were observed in the stained sections [6]. This confirms that the F98-induced tumor growth approximates the aggressive behavior of human GB. A reduced quantity of inoculated cells necessitates a greater number of divisions before the GB tumor achieves a size suitable for confirmation by MRI. It is generally understood that cell cultures have the potential to undergo modifications over time due to genetic instability, leading to changes in their phenotype [37]. Fortunately, we detected no microscopic differences between the different rat groups, signifying the preservation of phenotype across varying quantities of F98 cells. Moreover, this optimized F98 rat model can serve as a valuable platform to explore significant aspects of GB biology, including glioma cancer stem cells, and molecular markers like vascular endothelial growth factor (VEGF). VEGF is a key mediator of angiogenesis in GB, and the F98 model can be used to study its impact on tumor vascularization [38, 39]. The model also provides a first opportunity to study the behavior and characteristics of self-renewing glioma cancer stem cells, which play a vital role in disease progression and therapeutic resistance [40]. The newly optimized F98 rat model offers a comprehensive approach to studying angiogenesis and glioma stem cells in the context of GB and holds promise for potential breakthroughs in GB research and the development of therapies.

The final modification to minimize the extra-axial and extracranial tumor formations in the F98 GB rat model resides in the radiotherapy protocol. Recent developments of small animal micro-irradiators, such as the SARRP, enable accurate delivery of x-rays using kV photons. These devices are equipped with CT imaging for image guidance and accurate beam positioning [25]. In this study we pursued to mimic the clinical practice as closely as possible, keeping the balance between a clinically relevant and practically feasible animal-friendly treatment schedule. Therefore, we opted for a (hypo)fractionated radiotherapy approach, instead of a single dose as was performed by Bolcaen et al. and De Meulenaere et al. [21, 32], to further enhance the translatability from *in vivo* animal studies to clinical applications. Specifically, we administered three fractions of 9 Gy, a biologically equivalent dose to the 30 fractions of 2 Gy, the standard schedule applied in humans [37]. Bolcaen et al. previously introduced MRI-guided 3D conformal arc radiotherapy in the F98 GB rat model, using the SARRP to resemble the image-guided radiotherapy approach employed in clinical settings [32, 41, 42]. In our study, the divergence from Bolcaen et al. [32], lies in replacing one non-coplanar arc with a single static beam parallel to and following the inoculation route. This single static beam is further accompanied by two non-coplanar arcs, collectively irradiating the GB while minimizing exposure to healthy brain tissue [32]. This strategic inclusion of a single static beam aimed directly at the inoculation track serves to impede the potential migration of GB cells along this trajectory, thereby restraining the formation of extra-axial and extracranial tumors. In addition, the growth pattern of GB in the F98 GB rat model receiving fractionated radiotherapy in combination with TMZ chemotherapy was uniform for all animals, regardless of the amount of F98 GB cells. Tumor growth remained stable from day three to day eighteen, whereas exponential growth was observed between confirmation day and day three, as well as from day eighteen on. This confirms the existing, but also limited, efficacy of the standard of care for GB [43, 44]. Both Bolcaen et al. and De Meulenaere et al. demonstrated analogous outcomes when delivering a single dose of 20 Gy, although the suppression of tumor proliferation persisted only until day nine [21, 32]. A known consequence of radiotherapy is treatment-related edema, which can substantially impact patients’ quality of life [45, 46]. Fractionated radiotherapy delivers a series of small doses over a period of time, maximizing tumor cell damage while limiting adverse effects on healthy brain tissue [47]. This could explain the prolonged stabilization of GB growth in comparison to a high single dose, as the biological effect of fractionated radiation more closely approximates the clinical treatment schedule.

Considering the three modifications applied to the F98 GB rat model, none of the animals inoculated with 500, 1000, or 5000 F98 GB cells developed extracranial tumors, while extracranial tumors were observed in rats inoculated with 10 000 and 20 000 cells. Nevertheless, given the undesirability of extracranial tumors, it is prudent to favor an animal model devoid of this complication. Furthermore, increasing the number of inoculated cells from 500 to 20 000 F98 GB cells, yielded a tripling of the risk for extra-axial tumor formation. While statistical significance was not achieved, our results indicate a trend toward significance (p-value = 0.076). To further support this argument, we did demonstrate a significantly better overall survival of the rat groups inoculated with less than 10 000 F98 GB cells. Nevertheless, some degree of extra-axial tumor formation seems inevitable.

This study underscores a pivotal principle: the fewer the number of inoculated F98 cancer cells, the lower the risk of extra-axial and extracranial tumor formations. However, this risk reduction corresponds with increased variability concerning both the days until GB confirmation and the uniformity in GB growth. Our results suggest that inoculating 5000 F98 GB cells yields the most consistent outcomes. Hence, the rats inoculated with 5000 F98 GB cells, and treated with fractionated radiotherapy and concomitant TMZ chemotherapy, are chosen as recommended *in vivo* rat model for preclinical studies evaluating adjuvant therapies. Notably, this rat group did not develop extracranial tumors, and any extra-axial tumors that did manifest only emerged around day 21, with their volumes remaining comparatively small in relation to GB volumes. Taking these aspects into consideration, this F98 GB animal model now meets all of the above-mentioned criteria for a functionally effective brain tumor model. Additionally, the uniformity in GB growth in this group was akin to rats inoculated with 10 000 and 20 000 F98 GB cells. Moreover, this rat group had a survival probability of 100% until day 24, likely due to fewer occurrences of extra-axial and extracranial tumor formations, making survival in this model predictable. Tumor volumes measured by MRI showed a gradual increase in time, which parallels the pathological findings, thereby establishing the potential of survival time as an indirect indicator of tumor size [23]. Lastly, the variability in terms of days until GB confirmation was minimal and comparable to the rats inoculated with a higher amount of F98 GB cells.

In conclusion, for preclinical studies aimed at evaluating adjuvant therapies for GB, the F98/Fischer rat model, inoculated with 5000 cells, and treated with radiotherapy (three fractions of 9 Gy) and TMZ chemotherapy, emerges as an improved, reliable, and reproducible animal model.

## Supporting information

S1 Fig Visualisation of GB growth for each animal of the different experimental groups. (A) Rats inoculated with 500 F98 cells. (B) Rats inoculated with 1000 F98 cells. (C) Rats inoculated with 5000 F98 cells. (D) Rats inoculated with 10 000 F98 cells. (E) Rats inoculated with 20 000 F98 cells.
(TIF)

S1 Table Overview of humane endpoints for each animal. Rats were immediately euthanized when clinical and behavioral signs were observed.
(XLSX)
