# Supplementary figures and images for: An improved F98 glioblastoma rat model to evaluate novel treatment strategies incorporating the standard of care
Source: PLoS One. 2024 Jan 2;19(1):e0296360. doi: 10.1371/journal.pone.0296360 (PMC10760731; doi:10.1371/journal.pone.0296360)

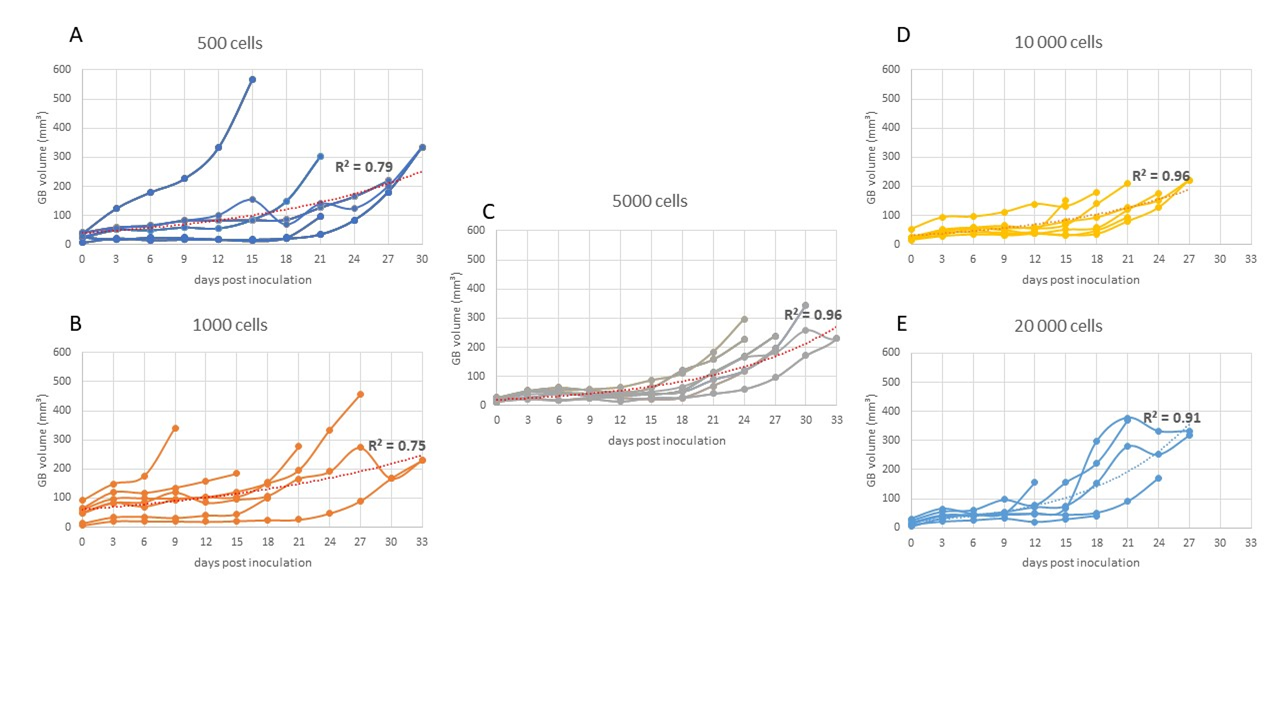

Supplement: S1 Fig — (A) Rats inoculated with 500 F98 cells. (B) Rats inoculated with 1000 F98 cells. (C) Rats inoculated with 5000 F98 cells. (D) Rats inoculated with 10 000 F98 cells. (E) Rats inoculated with 20 000 F98 cells. (TIF) [file pone.0296360.s001.tif]
